# Risk Factors for Moderate and Severe Persistent Pain in Patients Undergoing Total Knee and Hip Arthroplasty: A Prospective Predictive Study

**DOI:** 10.1371/journal.pone.0073917

**Published:** 2013-09-13

**Authors:** Patrícia R. Pinto, Teresa McIntyre, Ramón Ferrero, Armando Almeida, Vera Araújo-Soares

**Affiliations:** 1 Life and Health Sciences Research Institute (ICVS), School of Health Sciences, University of Minho, Braga, Portugal; 2 ICVS/3B’s – PT Government Associate Laboratory, Braga/Guimarães, Portugal; 3 Health Psychology Group, Newcastle University, Newcastle upon Tyne, United Kingdom; 4 Texas Institute for Measurement, Evaluation and Statistics (TIMES) and Department of Psychology, University of Houston, Houston, United States of America; 5 Alto Ave Hospital Center, Orthopedics Unit, Guimarães, Portugal; 6 Institute of Health & Society, Faculty of Medical Sciences, Newcastle University, Newcastle upon Tyne, United Kingdom; The University of Tokyo Hospital, Japan

## Abstract

Persistent post-surgical pain (PPSP) is a major clinical problem with significant individual, social and health care costs. The aim of this study was to examine the joint role of demographic, clinical and psychological risk factors in the development of moderate and severe PPSP after Total Knee and Hip Arthroplasty (TKA and THA, respectively). This was a prospective study wherein a consecutive sample of 92 patients were assessed 24 hours before (T1), 48 hours after (T2) and 4–6 months (T3) after surgery. Hierarchical logistic regression analyses were performed to identify predictors of moderate and severe levels of PPSP. Four to six months after TKA and THA, 54 patients (58.7%) reported none or mild pain (Numerical Rating Scale: NRS ≤3), whereas 38 (41.3%) reported moderate to severe pain (NRS >3). In the final multivariate hierarchical logistic regression analyses, illness representations concerning the condition leading to surgery (osteoarthritis), such as a chronic timeline perception of the disease, emerged as a significant predictor of PPSP. Additionally, post-surgical anxiety also showed a predictive role in the development of PPSP. Pre-surgical pain was the most significant clinical predictive factor and, as expected, undergoing TKA was associated with greater odds of PPSP development than THA. The findings on PPSP predictors after major joint arthroplasties can guide clinical practice in terms of considering cognitive and emotional factors, together with clinical factors, in planning acute pain management before and after surgery.

## Introduction

With the aging population, a significant rise in the prevalence of knee and hip osteoarthritis is expected and, consequently, an increase in the number of surgical interventions such as total knee arthroplasty (TKA) and total hip arthroplasty (THA). Being amongst the most commonly performed surgeries worldwide [1], [2], [3], these surgeries are aimed at reducing pain and disability, improving functional status and thus restoring quality of life [4], [5], [6], [7]. However, some patients may experience significant pain following surgery as well as scarce improvements in functional outcomes [8], [9]. Indeed, many patients experience moderate to severe levels of persistent post-surgical pain (PPSP) over the following months after arthroplasty, despite an absence of clinical or radiographic evidence of abnormalities [8], [9], [10]. This seems to highlight a potential influence of non-clinical factors, such as psychological factors, on the short and long-term outcomes of these types of surgeries. PPSP is a major clinical problem with significant individual, social and health care costs [11], [12], [13]. In studies focused on long-term outcomes following arthroplasties, attention has been mainly directed to potential predictors within demographic and clinical data [14], [15], [16], [17]. According to a recent systematic review [18] on studies seeking to explore psychological factors, the four most frequently assessed factors were mental health, patient expectations, anxiety and depression. Furthermore, pre-surgical mental health status and levels of pain catastrophizing have been reported as the most important predictors of pain after TKA and THA [7], [19], [20], [21].

Other potentially important but overlooked factors are patients’ illness representations as defined by the Common-Sense Self-Regulation Model (CS-SRM) [22], [23]. This model suggests that in the context of an illness, people tend to develop individual cognitive and emotional representations of their illness [24], [25], [26], enabling them to interpret and make sense of it [27]. These beliefs have been shown to explain significant variation in outcomes across a wide range of medical conditions and in response to different treatments [26], [28], [29], [30]. Previous studies using this theoretical perspective focused on the association between illness representations and functional activity, post-surgical adjustment or surgical recovery, rather than on their potential influence on post-surgical pain outcomes [27], [30], [31], [32]. Moreover, to date, with the exception of another study developed by our team regarding hysterectomy [33], no study has focused on the potential relationship between illness representations and the development of PPSP.

To our knowledge, studies aiming to understand the added contribution of psychological variables on PPSP have often missed the potential simultaneous influence of a multifactorial set of variables. Therefore, the aim of the present study is to explore, the combined contribution of demographic, clinical, and psychological factors as predictors of PPSP after knee and hip arthroplasties. Predictive models can potentially assist health care practitioners and patients in estimating the likelihood of success of major joint arthroplasties, providing clinicians with information that may be used to determine whether or not a patient is likely to develop moderate to severe levels of PPSP**.**

## Methods

### 1. Participants and Procedures

This was a prospective study, conducted in a central hospital in northern Portugal, wherein a consecutive sample of 130 patients with osteoarthritis was enrolled. Ethical approval was granted by the Alto Ave Hospital Centre Ethical Committee and all patients provided their written informed consent to participate in this study. Inclusion criteria were 40 to 80 years old, being able to understand written information (informed consent), without any psychiatric or neurologic pathology (e.g. psychosis, dementia), being classified with an ASA score (physical status classification of the American Society of Anesthesiologists) between grade I and III, and undergoing THA and TKA for diagnosis of coxarthrosis and gonarthrosis only (osteoarthrosis). Arthroplasties performed because of fractures were excluded, as well as hemiarthroplasties, revision and emergency arthroplasties.

**Figure 1:**
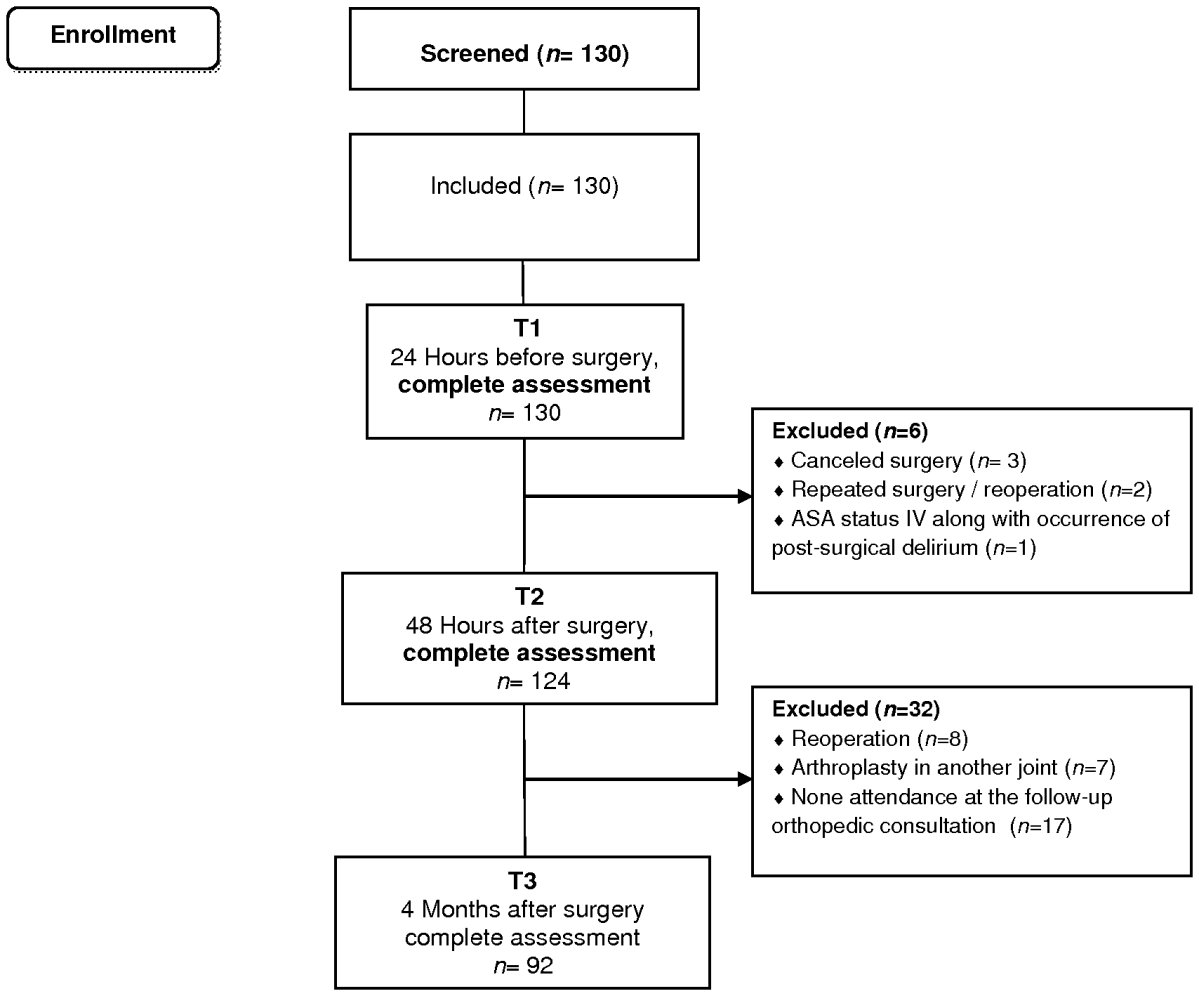
Flowchart of TKA and THA patients (screening, inclusion and assessment at all data points).

Patients were initially assessed 24 hours before (T1) and 48 hours after (T2) surgery, at the hospital setting (face to face). Follow-up assessment, also face to face, was performed in the consultations 4–6 months later, following a specific schedule for each outpatient consultation. **Figure 1** presents patient flow along the three assessment time points. From T1 to T2, 6 patients were withdrawn due to canceled surgery (*n* = 3), repeated surgery/reoperation (*n* = 2), and ASA status IV along with the occurrence of post-surgical delirium (*n* = 1). Of the remaining 124 patients assessed pre and post-surgery, 32 did not complete the 4–6 months follow-up assessment, leaving a sample of 92 patients for analyses including all time points. Those patients lost to follow up had post-surgical complications (e.g. infections) or accidents (prosthesis displacement) that required the performance of a revision arthroplasty in the operated joint (*n* = 8), underwent an arthroplasty in another joint (*n* = 7) or did not attend the follow-up orthopedic consultation (*n* = 17). The final sample consisted of 92 patients (61 women), with a mean age of 64.0±7.86 years.

### 2. Measures

Prior to the study, all instruments and study procedures were piloted in a similar sample of 12 patients for evaluation of their acceptability, feasibility and comprehensibility. Six THA and six TKA patients were submitted to surgery at the same hospital in which the present study was conducted, and presented similar socio-demographic and clinical characteristics to the study sample. Data from these 12 patients was used to refine the assessment protocol procedures as well as the assessment tools. The following questionnaires were administered in a face to face interview by a trained psychologist (see **Table 1**):

**Table 1:** Socio-demographic, clinical and psychological measures used at each assessment point T1, T2 and T3.

MEASURES	T1∶24 H beforesurgery	T2∶48 H aftersurgery	T3∶4/6 M after surgery
Socio-Demographic Questionnaire	**X**		**X**
Clinical Data	**X**	**X**	**X**
**BPI-SF:** Brief Pain Inventory – short form	**X**	**X**	**X**
**McGill Pain Questionnaire** (Frequency scale)		**X**	
**HADS:** Hospital Anxiety and Depression Scale	**X**	**X** (only anxiety)	**X**
**IPQ-R:** Revised Illness Perception Questionnaire (shortened version)	**X**

1. *Socio-Demographic Questionnaire*. It included questions on age, education, residence, marital status, professional status, household and parity.
2. *Clinical Questionnaire*. Consisted of questions about previous pre-surgical pain, its onset, duration and frequency, pain due to other causes, pain in other joints beyond the operated joint (specifically in knees and hips), back pain, disease onset, previous surgeries, height, weight, comorbidities, as well as the use of psychotropic drugs.
  1. Co-morbidities. Patients were directly questioned about the existence of pre-surgical co-morbid conditions that could affect TKA and THA surgical outcomes or this information was extracted from their medical chart. For this purpose, the Deyo–Charlson index [34] was used, consisting of a weighted scale of 17 comorbidities, such as hypertension, cardiac, pulmonary, renal and hepatic disease, diabetes mellitus, cancer, etc. The total number of co-morbid health conditions was added in order to calculate a total score. The weighting of severity that can be calculated when using this index was not used in our study. The summative score related to the total number of comorbid conditions was used, as done by others researchers [35], [36].
3. Brief Pain Inventory – short form (BPI-SF) [37]. It measured pain intensity on an 11-point numerical rating scale [Numerical Rating Scale (NRS) - from 0 or “no pain” to 10 or “worst pain imaginable” ]; it also measured pain analgesics, perception of analgesics relief, pain interference in daily activities (general activity, mood, walking, work, relations with others, sleep and enjoyment of life) and pain location. In this study, the internal consistency reliability [38] for the pain interference subscale scores was high (α = 0.88).
4. ‘Frequency scale’ of the McGill Pain Questionnaire [39]. Patients define their pain either as constant (continuous, steady), intermittent (periodic, rhythmic) or brief (momentary, transient). This subscale was used at T2 given that the characterization of a pain that is confined to a period of 48 hours cannot be described in terms of days, weeks or months, as was done for the assessment of pre-surgical pain at T1.
5. Hospital Anxiety and Depression Scale (HADS) [40]. The HADS consists of two 7-item sub-scales which measure anxiety (HADS-A) and depression (HADS-D) symptomatology amongst patients in non-psychiatric hospital settings. Item response format is a Likert scale ranging from 0 to 3. Sub-scale scores vary between 0 and 21. Higher scores represent higher levels of anxiety and depression. In the current sample, internal consistency reliability [38] was adequate for both anxiety (T1: α = 0.76; T2: α = 0.83) and depression (T1: α = 0.72).
6. Revised Illness Perception Questionnaire (IPQ-R) [41]. The IPQ-R assesses patient beliefs about osteoarthritis, the underlying condition that led to surgery. A psychometrically shortened version [42] was used with 7 subscales composed by 3 items each analyzing distinct dimensions of illness perceptions: “timeline acute/chronic” (α = 0.97; e.g. “My illness will last for a long time”); “personal control” (α = 0.79; e.g. “I have the power to influence my illness”); “treatment control” (α = 0.85; e.g. “Surgery can control my illness”); “illness coherence” (α = 0.87; e.g. “My illness is a mystery for me”); and “emotional representation” (α = 0.89; e.g. “When I think about my illness I get upset”). Timeline cyclical and consequences were not included due to their low reliability in this sample (α = 0.57 and α = 0.48, respectively). Items were rated on a 5-point adjective rating scale (1 = strongly disagree, 2 = disagree, 3 = neither agree nor disagree, 4 = agree, and 5 = strongly agree). To generate each subscale score, item scores were added, with subscale score range being 3–15. High scores reveal more maladaptive illness representations, with the exception of personal and treatment control subscales.
7. ‘Pain Catastrophizing scale’ of the *Coping Strategies Questionnaire – Revised Form* (CSQ-R) [43]. Pain catastrophizing was also assessed at T1. However this variable was not included in the statistical analyses due to missing data on 13 patients, which would substantially reduce power for the statistical analyses conducted.

### 3. Clinical Issues

Clinical data related to surgery, to anesthesia and to analgesia was gathered from medical records.

#### 3.1. Surgical procedure

From the sample of 92 patients, 44 (47.8%) were submitted to Total Knee Arthroplasty (14 on the left and 30 on the right side) and 48 (52.2%) to Total Hip Arthroplasty (25 on the left and 23 on the right side). Surgeries were performed by the team of Orthopedic Surgeons of the Orthopedics Unit of the above mentioned hospital.

A) Total Knee Arthroplasty (TKA)

For the knee, a cruciate-sacrifice prosthesis with a cobalt chromium bearing surface on an ultrahigh-molecular-weight polyethylene insert surface was placed in all cases. The surgical technique in all patients was an anterior midline approach with a medial parapatellar arthrotomy. These patients all had cruciate-sacrifice TKAs with all three components (tibial, femoral and patellar) cemented with a meticulous cement preparation technique. Resurfacing of the patellae was at the discretion of the surgeon. The most common technique for bone resection uses a 5° to 7° (depending on body habitus) valgus femoral cut and neutral tibial cut. Additionally, a correct ligament balancing was performed and tested to achieve equal and symmetric fixation and extension gaps. Intramedullary alignment guides were used for femoral and tibial cuts. The posterior cruciate ligament was resected. Bicondylar femoral and tibial components were implanted and cemented. A polyethylene liner was inserted between the metallic femoral and tibial prostheses. When at the infirmary, a continued passive range of motion was applied to these patients, who were also instructed to weight bear as tolerated.

B) Total Hip Arthroplasty (THA)

For hip patients, a press-fit technique was used for both components: femoral and acetabular. Supplemental screws were used to fix the cup, when necessary. Cobalt chromium on ultrahigh-molecular-weight polyethylene was the bearing surface in all cases. The surgical technique was quite similar in every case. All procedures were done through a direct antero-lateral approach (Watson-Jones) [44]. The choice of surgical approach was based upon surgeon preference given the clinical scenario (*i.e.*, body habitus, severity of disease, etc). In all cases, a cementless technique was performed with a tapered stem design (to interlock in the metaphysis with no diaphyseal fixation). Moreover, proximal porous coating was used to impart stability and allow for bone ingrowth. The implant was always collarless, allowing the prosthesis to be wedged into the bony metaphysis, providing for optimal fit and bone ingrowth. In addition, the tapered design allows subsidence into a tight fit and optimizes proximal load sharing of the implant, thereby optimizing bone ingrowth and minimizing stress shielding.

For both types of surgeries, anterior–posterior (AP) hip and lateral knee radiographs were taken and reviewed after surgery and before the patient was transferred to the infirmary for continued care. At the follow-up consultation (T3), radiographs were taken again. The radiographs were reviewed to ensure that the prosthesis was inserted properly and that alignment was correct, which was verified and confirmed for all the patients included in this sample.

After surgery, standardized postoperative nursing and physical therapy protocols were used for all patients. Patients were mobilized out of bed on the second postoperative day, and all patients had a postoperative anticoagulation protocol using LMWH (low-molecular-weight heparin). After surgery, patients were given systemic prophylactic antibiotics and prophylactic anticoagulant to decrease deep venous thrombosis risk.

Moreover, no research-related change was introduced in the standard clinical protocol.

#### 3.2. Anaesthetic technique

In all patients, the mode of anaesthesia was determined by the health care team according to the usual standard anaesthetic protocols at the hospital, with no research-related change being introduced.

The type of anesthesia in use was classified as: 1) loco-regional alone (*n* = 61/66.3%), which could be BSA (block spinal anaesthesia) or epidural, or as: 2) loco-regional (BSA or epidural) plus peripheral nerve blocks (*n* = 31/33.7%). ASA score (physical status classification of the American Society of Anesthesiologists) was recorded, including cases of ASA grade I (7/7.6%), II (67/72.8%) and III (18/19.6%).

#### 3.3. Analgesic protocols

All patients were prescribed a standardized analgesia protocol according to the usual standard norms of care at the hospital. This protocol, determined and supervised by the Acute Pain Unit, led by an Anesthesiologist, was instituted still in the recovery room, prior to patient transfer to the orthopedic infirmary.

Delivery of the analgesic protocol could be intravenous, epidural or peri-neural, followed by oral analgesics on subsequent days.

The standardized intravenous protocol was composed by a continuous intravenous infusion (DIB - delivered infusion balloon) of tramadol (600 mg), metamizol (6 g) and metoclopramide (60 mg). The standardized epidural protocol was a continuous epidural infusion (DIB) with ropivacaine (0.1%) and fentanyl (3 ug/ml). Finally, the standardized peri-neural protocol included a continuous peri-neural infusion (DIB) with ropivacaine (0.1%). For the three types of protocols, Paracetamol (1 g 6/6 h) and Non-steroidal anti-inflammatory drugs (NSAIDS - ketorolac 30 mg 12/12 h or parecoxib 40 mg 12/12 h) were always included as coadjuvant analgesics. All analgesic regimens included prokinetic treatment that was standardized to metoclopramide (10 mg i.v. 8/8 h). All protocols had indications for the prescription of rescue analgesics beyond the standardized analgesic protocol given moderate to severe acute post-surgical pain levels (NRS>3) [45], [46]. Acute pain team professionals were blind to patient’s participation in the study.

### 4. Outcome Measure – Pain 4–6 Months after TKA and THA

The outcome measure was reporting moderate to severe pain 4–6 months after surgery (PPSP). Patients reporting significant “worst pain” levels in the surgical area (NRS >3) were considered as being PPSP positive, similarly to previous studies [8], [47], [48]. There are several reasons for this option. First, this cut-off was based on previous recommendations considering the differential impact of pain levels above 3 [45], [46], [49], [50]. Second, it is assumed that in face of these major surgeries, 4–6 months after, mild levels of pain can still occur without necessarily implying pain chronification [10], [51]. Third, we are assessing pain in terms of worst pain levels. Given that major joints, such as the knee and hip, take some time to heal, and are particularly affected by movement, it is usually recognized by patients that pain during movement or activities that require more physical effort corresponds to “worst pain” [10], [45], [49], [50]. Studies on pain after total joint arthroplasties usually measure the improvements in mean pain scores from the pre-surgical period to the various post-surgical follow ups. Nevertheless, as argued by Beswick and colleagues [52], the mean pain score at each time point has a concomitant standard deviation, which implies that certain pain patients are not adequately represented. By considering “worst pain” score as a pain outcome, this shortcoming has the potential of being circumvented.

### 5. Statistical Analyses

Data were analyzed using the Statistical Package for the Social Sciences (SPSS version 19.0). Internal consistency of responses to the questionnaires was assessed using Cronbach’s alpha [38] (see above).

The primary outcome variable was the report of moderate to severe pain at the 4 to 6 months follow-up. Patients were classified into two groups: none or mild pain (NRS ≤3 for “worst pain level”) and moderate to severe pain (NRS>3 for “worst pain level”) group.

Normality for continuous variables was assessed with the Kolmogorov-Smirnov Test evidencing that distribution of data differed significantly from normality assumptions. Thus, non-parametric Mann-Whitney or Chi-square tests (*χ2*) were performed to compare socio-demographic, clinical and psychological measures between these two pain groups (NRS≤3 vs NRS>3) and also between the two surgical groups (TKA and THA). Descriptive statistics for continuous variables are presented as mean and standard deviations, whereas categorical data are presented as numbers and percentages.

Hierarchical logistic regression analyses were conducted to determine risk factors for moderate to severe levels of PPSP. This approach enters all independent variables into the regression equation, but sequentially in blocks of independent variables. This allows testing if a given block of variables accounts for additional variability in the dependent variable, over and above the independent variables added to the model in previous blocks.

The potential predictors selected for inclusion were those found to better distinguish between the two pain groups (*p*<0.05). Those were conceptualized into three categories: demographic, clinical and psychological. In addition, Spearman correlations were calculated for the psychological variables that distinguished between NRS≤3 and NRS>3 groups in order to further investigate the association between psychological constructs. In all comparisons, two-sided tests were performed with *p*<0.05 used to indicate statistical significance.

Two different hierarchical logistic regression models were used to analyze the factors associated with the occurrence of moderate to severe levels of PPSP. One of the models centered on pre-surgical factors (T1) and the second one addressed the immediate post-surgical period (T2). For both models, “Pre-surgical pain intensity” and “Number of pain problems elsewhere” were entered in block 1. Another clinical variable, pre-surgical pain interference, was considered for inclusion in block 1. Nevertheless, it showed considerable overlap to the other two baseline pain predictors (“Pre-surgical pain intensity” and “Number of pain problems elsewhere”) and was excluded from both models due to multicollinearity (VIF >2). Type of arthroplasty was entered in block 2 as it distinguished the two pain groups in univariate analyses as found in previous studies [18]. Demographic factors, such as sex or age, did not differentiate pain groups and thus were not considered as potential predictors in the regression models. Psychological factors that distinguished the two groups in T1 and T2 assessments were included in the subsequent blocks. In Model 1, “pre-surgical anxiety” was entered in block 3 and illness perceptions, such as “timeline acute/chronic perception of the condition that led to surgery” and “emotional representation of the condition that led to surgery” were added to the equation in block 4. In the second regression model, focused on acute post-surgical (T2) predictors of PPSP, “acute post-surgical pain” was entered in block 3 and “post-surgical anxiety” was entered in block 4.

In all regression models, the variance inflation factor value (VIF) for every independent variable was calculated to control for the influence of multicollinearity, with each variable only being included if its VIF was less than 2.

## Results

### 1. Pain 4 to 6 Months after TKA and THA: Socio-demographic, Clinical and Psychological Characteristics by Group (None or Mild vs. Moderate to Severe pain)

Fifty-four patients reported none or mild pain (NRS ≤3) 4–6 months after arthroplasty, whereas 38 reported moderate to severe pain (NRS >3). **Table 2** shows socio-demographic and clinical characteristics of both the total patient sample and each of the post-surgical pain severity groups (NRS ≤3 and NRS >3). Regarding the impact of the specific type of arthroplasty, TKA was more significantly associated with moderate to severe pain than THA (*χ2* = 8.372, *p* = 0.004). The groups did not differ significantly in any of the socio-demographic measures. Moreover, they did not differ in any other clinical measure with the exception of worst level of pre-surgical pain intensity (*z* = −2.405, *p* = 0.016), pre-surgical pain interference (*z* = −2.115, *p* = 0.034) and number of pain problems elsewhere (*z* = −2.392, *p* = 0.017), with patients presenting moderate to severe PPSP reporting worst results. Furthermore, moderate to severe PPSP patients showed a worst pre-surgical psychological profile (**Table 2**), revealing more anxiety (*z* = −2.166, *p* = 0.030), perception of more chronicity of the medical condition (osteoarthritis) that led to surgery (“Timeline acute/chronic”: *z* = −2.607, *p* = 0.009), along with a more negative emotional representation of the surgical condition (*z* = −2.943, *p* = 0.003).

**Table 2:** Sample characteristics and results for non-parametric group comparison tests (Mann-Whitney and Chi-Square) between pain groups (T3), on socio-demographic, clinical and psychological measures at T1 and T2.

MEASURES	Total (*N = *92)	Absence or mild PPSPNRS≤3 (*n = *54)	Moderate to severe PPSP NRS>3 (*n* = 38)	*Z/χ2*	*p*
**Type of arthroplasty -** TKA	44 (47.8%)	19 (35.2%)	25 (65.8%)	8.372	***0.004***
**Patient baseline characteristics – T1**					
Socio-demographic: Age (years)	64.0 (7.86)	64.5 (7.77)	63.3 (8.04)	−1.064	*0.287*
Socio-demographic: Sex (women)	61 (66.3%)	34 (63.0%)	27 (71.1%)	0.653	*0.419*
***Clinical – pre-surgical general indicators***					
Disease onset (months)	111 (115)	101 (101)	125 (133)	−0.494	*0.621*
BMI^1^ (Kg/m2)	29.4 (4.66)	29.3 (4.65)	29.5 (4.75)	−0.253	*0.800*
Previous surgeries (yes)	80 (87%)	45 (83.3%)	35 (92.1%)	1.513	*0.219*
Comorbidities total^2^	2.15 (1.24)	2.15 (1.23)	2.16 (1.26)	−0.061	*0.951*
**Clinical - pre-surgical pain indicators**					
Intensity^3^ (worst level)	6.87 (1.94)	6.47 (1.99)	7.42 (1.73)	−2.405	***0.016***
Intensity^3^ (average level)	4.48 (1.24)	4.34 (1.16)	4.68 (1.34)	−1.061	*0.288*
Duration (>2 years)	73 (79.3%)	41 (75.9%)	32 (84.2%)	0.934	*0.334*
Pain Total Interference^4^ (0–70)	27.3 (12.3)	24.8 (13.5)	30.8 (9.30)	−2.115	***0.034***
Nr. of pain problems elsewhere	1.29 (1.36)	1.04 (1.26)	1.67 (1.43)	−2.392	***0.017***
Pain in other joints (yes)	33 (35.9%)	17 (31.5%)	16 (42.1%)	1.094	*0.296*
Back pain (yes)	45 (48.9%)	24 (44.4%)	21 (55.3%)	1.045	*0.307*
**Pre-surgical psychological variables**					
**HADS** a **:** Anxiety	5.18 (3.98)	4.41 (3.69)	6.29 (4.16)	−2.166	***0.030***
**HADS** a **:** Depression	2.40 (3.16)	2.06 (3.07)	2.89 (3.27)	−1.585	*0.113*
**IPQ – R** b **:** Timeline acute/chronic	8.38 (2.82)	7.76 (2.62)	9.26 (2.88)	−2.607	***0.009***
**IPQ – R** b **:** Personal control	6.50 (2.13)	6.17 (1.73)	6.97 (2.53)	−1.056	*0.291*
**IPQ – R** b **:** Treatment control	12.0 (1.11)	11.9 (1.09)	12.1 (1.16)	−0.132	*0.895*
**IPQ – R** b **:** Illness coherence	7.66 (3.11)	7.61 (3.07)	7.74 (3.22)	−0.020	*0.984*
**IPQ – R** b **:** Emotional representation	9.38 (3.12)	8.50 (3.36)	10.6 (2.26)	−2.943	***0.003***
**Post-surgical data 48H after surgery-T2**					
Type of anesth.^5^: loco-regional+PNB	31 (33.7%)	16 (29.6%)	15 (39.5%)	0.967	0.325
Post-surgical pain intensity^3^– worst	6,50 (2.51)	5.94 (2.42)	7.29 (2.45)	−2.513	**0.012**
Post-surgical pain intensity^3^- average	3.78 (1.46)	3.36 (1.51)	4.36 (1.17)	−3.137	**0.002**
Pain Frequency^6^: constant	49 (53.3%)	25 (46.3%)	24 (63.2%)	2.547	*0.110*
Rescue analgesia (yes)	39 (42.4%)	19 (35.2%)	20 (52.6%)	2.780	*0.095*
HADS^a^: Anxiety	3.58 (3.46)	2.76 (3.26)	4.74 (3.45)	−3.062	**0.002**
Length of hospital stay (days)	7.01 (2.7)	6.55 (1.53)	7.66 (3.72)	−1.566	0.117

Note. Continuous variables are presented as median (range); categorical variables are presented as *n* (%); T1–24 hours before surgery; T2–48 hours after surgery; T3–4–6 months after surgery;

1 BMI = body mass index;

2 Comorbidities total = number of comorbid health conditions;

3 NRS(BPI) = Numerical Rating Scale 0–10 from Brief Pain Inventory;

4 Pain Total Interference Scale 0–70 from Brief Pain Inventory (BPI);

5 Type of anesthesia: Anesthesia loco-regional alone: BSA or epidural vs Anesthesia loco-regional (BSA or epidural)+peripheral nerve blocks (PNB);

6 Pain Frequency: constant pain vs intermittent or brief pain, assessed via frequency subscale of McGill Pain Questionnaire;

a HADS = Hospital Anxiety and Depression Scale;

b IPQ-R = Illness Perception Questionnaire-Revised.

Patients reporting moderate to severe levels of PPSP at T3 also reported high anxiety (*z* = −3.062, *p* = 0.002) and a heightened acute post-surgical pain intensity, both in terms of average (*z* = −3.137, *p* = 0.002) and worst (*z* = −2.513, *p* = 0.012) pain, at T2 (48 hours after surgery). No other distinction on clinical parameters was found between groups at T2 (e.g. type of anesthesia, length of stay, rescue analgesia or pain frequency).

### 2. Differences between Patients Submitted to THA and TKA on Socio-demographic, Clinical and Psychological Measures at T1, T2 and T3

At T1, regarding baseline measures, arthroplasty groups did not differ on any socio-demographic characteristic (see Table 3), except for age and sex. Patients undergoing TKA were older than those undergoing THA (*z* = −2.364, *p* = 0.018), and were also comprised by more women than men (*χ2* = 4.541, *p* = 0.033). Both groups were similar concerning clinical measures, such as BMI (Body Mass Index) and previous surgical procedures. However, TKA patients had suffered longer from their surgical disease (*z* = −2.344, *p* = 0.019) and presented more medical comorbidities (*z* = −2.052, *p* = 0.040). Although the groups did not differ in terms of pre-surgical pain intensity and in total pre-surgical pain interference levels on daily activities, TKA patients reported pre-surgical pain of longer duration (*χ2* = 6.879, *p* = 0.009). Furthermore, this latter group presented more pain problems elsewhere (*z* = −2.857, *p* = 0.004), as well as pain in other joints (*χ2* = 5.155, *p* = 0.023) (see **Table 3**).

**Table 3:** Results for non-parametric group comparison tests (Mann-Whitney and Chi-Square) between THA and TKA patients on socio-demographic, clinical and psychological measures at T1, T2 and T3.

MEASURES	THA (*n = *48)	TKA (*n = *44)	*Z/χ2*	*p*
**Patient baseline characteristics – T1**				
*Socio-demographic:* Age (years)	62.0 (8.05)	66.2 (7.10)	−2.364	***0.018***
*Socio-demographic:* Sex (women)	27 (56.3%)	34 (77.3%)	4.541	***0.033***
***Clinical – general indicators***				
Disease onset (months)	81.9 (75.8)	141.6 (140.2)	−2.344	***0.019***
BMI^1^ (Kg/m2)	28.9 (4.39)	29.8 (4.95)	−0.714	*0.475*
Previous surgeries (yes)	40 (83.3%)	40 (90.9%)	1.162	*0.281*
Comorbidities total^2^	1.92 (1.32)	2.41 (1.11)	−2.052	***0.040***
**Clinical - pre-surgical pain indicators**				
Intensity^3^ (worst level)	6.91 (2.06)	6.82 (1.82)	−0.182	*0.855*
Intensity^3^ (average level)	4.47 (1.08)	4.50 (1.41)	−0.129	*0.898*
Duration (>2 years)	33 (68.8%)	40 (90.9%)	6.879	***0.009***
Pain Total Interference^4^ (0–70)	26.9 (12.8)	27.7 (11.8)	−0.149	*0.882*
Nr. of pain problems elsewhere	0.87 (1.10)	1.69 (1.46)	−2.857	***0.004***
Pain in other joints (yes)	12 (25.0%)	21 (47.7%)	5.155	***0.023***
Back pain (yes)	20 (41.7%)	25 (56.8%)	2.109	*0.146*
**Psychological variables**				
**HADS** a **:** Anxiety	4.75 (3.89)	5.66 (4.06)	−1.205	*0.228*
**HADS** a **:** Depression	2.46 (3.31)	3.82 (3.55)	−0.172	*0.864*
**IPQ – R** b **:** Timeline acute/chronic	8.17 (2.70)	8.61 (2.95)	−0.731	*0.465*
**IPQ – R** b **:** Timeline cyclical	9.06 (2.44)	8.89 (2.46)	−0.565	*0.572*
**IPQ – R** b **:** Consequences	10.3 (2.42)	10.3 (2.28)	−0.261	*0.794*
**IPQ – R** b **:** Personal control	6.00 (1.52)	7.05 (2.54)	−1.746	*0.081*
**IPQ – R** b **:** Treatment control	11.8 (1.25)	12.2 (0.92)	−1.349	*0.177*
**IPQ – R** b **:** Illness coherence	8.38 (3.43)	6.89 (2.54)	−2.090	***0.037***
**IPQ – R** b **:** Emotional representation	9.00 (3.22)	9.80 (2.99)	−1.236	*0.217*
**Postsurgical data 48H after surgery-T2**				
Post-surgical pain intensity^3^– worst	5.90 (2.62)	7.16 (2.23)	−2.275	**0.023**
Post-surgical pain intensity^3^- average	3.39 (1.46)	4.19 (.37)	−2.453	**0.014**
Pain Frequency^5^: constant	21 (43.8%)	28 (63.6%)	3.647	*0.056*
Rescue analgesia (yes)	16 (33.3%)	23 (52.3%)	3.372	*0.066*
HADS^a^: Anxiety	3.35 (3.40)	3.82 (3.55)	−0.589	0.556
Length of hospital stay	6.77 (1.81)	7.28 (3.45)	−0.221	0.825
**Postsurgical data 4–6 M after surgery-T3**				
Post-surgical pain intensity^3^– worst	2.01 (1.94)	3.75 (2.25)	−3.638	**<0.001**
Post-surgical pain intensity^3^- average	1.36 (1.34)	2.59 (1.47)	−3.863	**<0.001**

Note. Continuous variables are presented as median (range); categorical variables are presented as *n* (%); T1–24 hours before surgery; T2–48 hours after surgery; T3–4–6 months after surgery;

1 BMI = body mass index;

2 Comorbidities total = number of comorbid health conditions;

3 NRS(BPI) = Numerical Rating Scale 0–10 from Brief Pain Inventory;

4 Pain Total Interference Scale 0–70 from Brief Pain Inventory (BPI);

5 Pain Frequency: constant pain vs intermittent or brief pain, assessed via frequency subscale of McGill Pain Questionnaire;

a HADS = Hospital Anxiety and Depression Scale;

b IPQ-R = Illness Perception Questionnaire-Revised.

The two arthroplasty groups did not differ in any of the psychological baseline measures, with the exception of illness coherence, which was lower among THA patients (*z* = −2.090, *p* = 0.037) (**Table 3**); for the THA group, the surgical illness made less sense than for the TKA group. Moreover, at T2 (48 hours post-surgery) the groups did not show any significant difference on psychological factors, such as in anxiety. In conclusion, at baseline the groups were mostly homogeneous in terms of their psychological profile and this was maintained at T2 for anxiety.

Immediately after surgery, TKA patients exhibited heightened acute post-surgical pain intensity, both in terms of average (*z* = −2.453, *p*<0.001) and worst (*z* = −2.275, *p*<0.001) pain. No other distinction on clinical parameters was found between groups 48 hours after surgery (e.g. length of stay, rescue analgesia or pain frequency).

### 3. Pre-surgical (T1) Risk Factors for PPSP 4 to 6 Months after TKA and THA

The results of the hierarchical logistic regression for Model 1 are presented in **Table 4**. All variables included emerged as significant at their specific block, with the exception of pre-surgical anxiety (block 3) and pre-surgical emotional representation (block 4). In the final model, pre-surgical pain intensity and type of surgery emerged as significant clinical factors for developing moderate to severe PPSP and pre-surgical representations regarding the duration of the illness as significant psychological predictors. More specifically, patients undergoing major joint arthroplasties complaining of higher levels of pre-surgical pain intensity prior to surgery, present a higher likelihood of developing moderate to severe PPSP (OR = 1.347, 95% CI, 1.005–1.806). Furthermore, patients who are submitted to TKA reveal higher risk of developing PPSP (OR = 4.490, 95% CI, 1.473–13.691) when compared to THA patients. The psychological predictor “pre-surgical illness perceptions of illness duration” (regarding osteoarthritis, the condition leading to surgery), namely a chronic perception of the surgical disease, seems to have a significant predictive role in PPSP after major joint arthroplasty (OR = 1.337, 95% CI, 1.064–1.679). Another illness perception, “pre-surgical emotional representation” was a marginally significant predictor (OR = 1.234, 95% CI, 0.990–1.537), with patients who have a more negative emotional perception of the illness presenting higher risk of PPSP development. It is noteworthy that the variables “pre-surgical anxiety” and “number of pain problems elsewhere” did not show any predictive value in the final model, despite the fact that both significantly distinguished the two pain groups, as evidenced by univariate analysis. No collinearity problems were identified regarding these variables and the other predictors.

**Table 4:** Model 1 - Hierarchical logistic regression analysis of Moderate to Severe Persistent Post-surgical Pain 4–6 months (T3) following TKA and THA on clinical and psychological measures at baseline (T1).

Model 1	Wald	*OR* (95% CI)	*p*
**Block 1**			
Pre-surgical pain intensity^1^	4.294	1.300 (1.014–1.666)	**0.038**
Nr. Of pain problems elsewhere^2^	5.003	1.491 (1.051–2.116)	**0.025**
**Block 2**			
Type of surgery (TKA)^3^	6.193	3.625 (1.315–9.994)	**0.013**
**Block 3**			
Pre-surgical anxiety^a^	2.693	1.124 (0.977–1.294)	0.101
**Block 4 (Final Model)**			
Pre-surgical pain intensity^1^	3.971	1.347 (1.005–1.806)	**0.046**
Nr. Of pain problems elsewhere^2^	0.006	0.984 (0.647–1.496)	0.939
Type of surgery (TKA)^3^	6.973	4.490 (1.473–13.691)	**0.008**
Pre-surgical anxiety^a^	0.907	1.081 (0.921–1.269)	0.341
Pre-surgical timeline acute/chronic^b^	6.235	1.337 (1.064–1.679)	**0.013**
Pre-surgical emotional representation^b^	3.496	1.234 (0.990–1.537)	*0.062*

Note. After removing 2 outliers, this final model correctly predicted 74% of all patients; *χ2*(6) = 31.696; *p*<0.001; Nagelkerke *R* ^2^ = 0.412; OR = odds ratioCI = confidence interval; bold = significant at *p*≤.05; italics = marginally significant *p*≤.10.

1 Continuous variable, NRS - Numerical Rating Scale (0–10) from BPI-SF: Brief Pain Inventory-Short Form;

2 Continuous variable;

3 Dichotomic variable: 0 = THA: Total Hip Arthroplasty; 1 = TKA: Total Knee Arthroplasty;

a Continuous variable, HADS-A: Hospital Anxiety and Depression Scale - anxiety subscale;

b Continuous variables, subscales of IPQ-R: Illness Perception Questionnaire Revised.

### 4. Post-surgical (T2) Risk Factors for PPSP 4 to 6 Months after TKA and THA

Model 2 results address post-surgical predictors (T2) of PPSP over and above the same demographic and clinical variables used for Model 1 (see **Tables 4** **and** **5**). The results for pre-surgical predictors replicated those of Model 1. Acute post-surgical pain intensity (block 3) did not yield significant results. On the other hand, post-surgical anxiety (block 4) was a significant predictor of moderate to severe PPSP (OR = 1.250, 95% CI, 1.045–1.495) at 4 to 6 months follow-up (**Table 5**).

**Table 5:** Model 2 - Hierarchical logistic regression analysis of Persistent Post-surgical Pain 4–6 months (T3) following TKA and THA on demographic and clinical baseline measures (T1), and post-surgical pain and anxiety 48 h after surgery (T2).

Model 3	Wald	*OR* (95% CI)	*p*
**Block 1**			
Pre-surgical pain intensity^1^	4.736	1.324 (1.028–1.704)	**0.030**
N° of pain problems elsewhere^2^	5.321	1.514 (1.064–2.155)	**0.021**
**Block 2**			
Type of surgery (TKA)^3^	6.514	3.840 (1.367–10.793)	**0.011**
**Block 3**			
Post-surgical pain intensity^1^	2.553	1.191 (0.961–1. 475)	0.110
**Block 4 (Final Model)**			
Pre-surgical pain intensity^1^	6.829	1.502 (1.107–2.038)	**0.009**
N° of pain problems elsewhere^2^	0.751	1.195 (0.798–1.790)	0.386
Type of surgery (TKA)^3^	7.102	4.842 (1.518–15.444)	**0.008**
Post-surgical pain intensity^1^	0.244	1.063 (0.835–1.352)	0.621
Post-surgical anxiety^a^	5.990	1.250 (1.045–1.495)	**0.014**

Note. After removing 2 outliers, this final model correctly predicted 75% of all patients; *χ2*(5) = 27.935 *p*<0.001; Nagelkerke *R* ^2^ = 0.372; OR = odds ratio; CI = confidence interval; bold = significant at *p*≤.05.

1 Continuous variable, NRS - Numerical Rating Scale (0–10) from BPI-SF: Brief Pain Inventory-Short Form;

2 Continuous variable;

3 Dichotomic variable: 0 = THA: Total Hip Arthroplasty; 1 = TKA: Total Knee Arthroplasty;

a Continuous variable, HADS-A: Hospital Anxiety and Depression Scale - anxiety subscale.

## Discussion

The present study aimed to examine the joint role of demographic, clinical and psychological risk factors for persistent pain experience 4 to 6 months after total knee (TKA) and hip arthroplasty (THA). Amongst the clinical factors, pre-surgical pain intensity and type of arthroplasty were the key predictors of PPSP development. Regarding psychological variables, pre-surgical illness perceptions concerning the duration (acute versus chronic) of the condition leading to surgery, namely osteoarthritis, arose as a significant predictive factor. Post-surgical anxiety was the second psychological variable found to be a risk factor for the development of moderate to severe PPSP. The results of this study improve knowledge on PPSP after major joint arthroplasties, and point to potential preventive targets for healthcare professionals.

### 1. Predictors of PPSP after TKA and THA

#### 1.1. Clinical predictors

In line with previous evidence, either in arthroplasties [6], [8], [53], [54] or in other surgical procedures [55], [56], [57], [58], pre-surgical pain intensity emerged as a significant PPSP predictor. In the current study all patients reported pre-surgical pain, albeit with variations in its intensity, since pain is the primary reason for undergoing arthroplasty [10], [59]. Evidence has shown that prolonged pain stimulation exacerbates the nociceptive system, causing peripheral and central sensitization of both nociceptors and central nervous system neurons [60]. The association between the presence of pre-surgical pain and PPSP may thus be explained by the pre-surgical occurrence of plastic changes in the nociceptive system and supraspinal pain control system [61], [62], [63] that are associated with a continuous and repetitive pain stimulation.

This study did not corroborate previous findings wherein acute post-surgical pain arose as a PPSP predictor [57], [64], [65], [66], [67]. Concerning arthroplasties, we are aware of only two studies [15], [68] wherein post-surgical pain predicted PPSP after THA and TKA, although that measure was assessed retrospectively, recalled 1 year after surgery. In the other arthroplasty studies, the trend has been similar to current data: pre-surgical pain showing a stronger predictive value of PPSP when compared to acute post-surgical pain [6], [8], [53], [54]. In our study, 80% of patients had pre-surgical pain for more than 2 years (chronic pain). Thus, it seems plausible that in face of long-term pre-surgical pain, it would be its regular intensity, rather than the short-term post-surgical pain intensity, that would be determinant in the neuro-physiologic processes underlying PPSP development. Through quantitative sensory testing, some osteoarthritis patients have shown central sensitization, exhibiting reduced pain thresholds, in several body areas [69], [70], [71]. Central hypersensitivity could explain the less favorable results amongst arthroplasty patients who report high pre-surgical pain intensity [54], suggesting that the degree of previous central sensitization is a crucial determinant of surgery, and thus, an element of pre-surgical prognostic value. These results suggest that arthroplasty patients who are screened with higher pre-surgical pain intensity need to be targeted and offered special care in terms of pre-surgical intervention focused on effective pain management. Additionally, they may benefit from the prescription of more aggressive perioperative analgesic protocols, previously shown to be efficient in PPSP prevention [72].

As expected [73], [74], [75], patients submitted to TKA revealed higher odds of developing PPSP, when compared to THA patients. It has been suggested [10] that these PPSP differences cannot be attributed to demographic differences between patients undergoing TKA and THA, but result instead from several pathological sources, such as the subcutaneous nature of the joint or its nerve supply or kinematics. It has also been hypothesized that psychosocial factors may be linked to these differences [10]. Although the present study reveals some demographic and pre-surgical clinical differences between the two groups, as well as distinctive acute post-surgical pain levels, it does not show significant differences in the baseline psychological profile. Pathophysiological reasons seem thus the most likely factors accounting for these distinct outcomes, although future research may shed some light on this issue.

#### 1.2. Psychological predictors

Concerning the influence of psychological factors on PPSP, one interesting finding of this study is that in face of the prospect of undergoing an arthroplasty, patients who perceive the surgical disease (osteoarthritis) as more chronic are also more likely to develop moderate to severe PPSP.

Timeline beliefs concern the patient’s expectations about the duration of the disease and its characteristic course [76] whether it is acute (short-term) or chronic (long-term) [77]. Evidence has shown that the perception of chronicity associated to medical conditions is related with higher levels of functional limitations, in distinct diseases [30], [31], [78], [79], [80], [76], [81]. Concerning specifically arthroplasties, Orbell and colleagues [27] investigated the role of illness representations on functional activity, but not on pain outcomes. The perception that one illness will be of long-term duration has important implications in the way patients effectively feel that they can manage it [82]. Many patients view osteoarthritis as a normal part of aging [83] and, consequently, are more likely not to act proactively to manage pain or surgical recovery.

Additionally, the emotional illness representations did retain marginal significance, needing to be revisited in further studies. Other studies have demonstrated that patients’ beliefs about whether their illness has an emotional impact, such as feeling depressed, angry or upset, relate with health outcomes [31], [78], [76].

Although all these studies demonstrated the significant role of illness representations in the prediction of health and disease-related outcomes, none have tried to relate the former with pain outcomes, with the exception of previous studies performed by our team, one concerning PPSP after hysterectomy [33] and the other focused on acute post-surgical pain after major joint arthroplasties [36]. Hence, this is the first study testing illness representations as potential risk factors for PPSP after major joint arthroplasties.

Present findings suggest that pre-surgical arthroplasty patients could benefit from pre-surgical preventive interventions, aimed at restructuring illness cognitions, in order to reduce the likelihood of PPSP development. This can be achieved using brief cognitive-behavior techniques, involving the identification of maladaptive illness representations and the promotion of adaptive cognitions concerning the surgical disease (such as regarding illness duration). This often involves the reframing of illness perceptions and the induction of a more positive view of the expectations concerning the surgical disease [24], [26].

A major focus of our work was to identify the predictors of persistent pain after TKA and THA surgery. Both emotional and cognitive factors emerged as important predictors of PPSP. Previous research corroborated the role of pre-surgical anxiety as a potential risk factor for PPSP [8], [33], [84], [85], [86], whereas post-surgical anxiety has not been explored as a potential predictor. Present findings revealed that pre-surgical anxiety did not yield significant results in the prediction of PPSP after major joint arthroplasties. Instead, post-surgical anxiety emerged as a significant predictor, further supporting the idea that post-surgical anxiety might be a more accurate predictor of PPSP than pre-surgical anxiety. It is somewhat surprising that, with the exception of a study by our team [33], wherein post-surgical anxiety was shown to predict PPSP development after hysterectomy, anxiety after surgery had not been studied as a potential PPSP predictor. Emotional factors seem to play a crucial role in the establishment of persistent post-surgical pain, regardless of the type of surgery and surgical assessment point. Furthermore, post-surgical anxiety is more proximal in time to persistent pain than pre-surgical anxiety.

Even though present findings revealed a stronger predictive value of post-surgical anxiety in comparison to pre-surgical anxiety, the latter has been found to be a strong predictor of the former, in orthopaedic samples and in across other surgeries [45], [86], [34]. This suggests that early intervention on pre-surgical anxiety could benefit anxiety after surgery. Brief cognitive-behavior techniques such as relaxation, imagery, reassurance and positive coping self-statements [87], [88], [89], [90] can benefit patients both before surgery and during hospitalization. Present findings also indicate that post-surgical anxiety management needs to be considered an important element of the post-surgical protocol, which is strategically targeted to reduce the likelihood of PPSP development after joint arthroplasties. Further research needs to test the impact of these types of interventions on patient post-surgical clinical and psychological outcomes.

### 2. Limitations

The aim of the current study was to approach TKA and THA jointly, as both are categorized as major joint surgeries. However, we are aware that different results have been reported for PPSP after TKA and THA, regarding both the influence of psychological factors affecting PPSP [18] and PPSP prevalence, which is higher in TKA [4], [74]. Although we controlled for type of surgery in the statistical analyses and also identified the distinct features and statistical significant differences between the two surgical procedures, the generalizability of the findings across surgeries needs to be interpreted with caution.

Another potential limitation concerning internal validity is the loss to follow-up of 32 patients from T2 to T3. Additional analyses were performed to investigate potential differences between the patients that remained in the study and those who were not assessed at follow ups but no significant differences were found for baseline characteristics (T1) or in respect to acute post-surgical issues (T2). Therefore, the 92 remaining patients seem to be representative of this cohort.

Regarding external validity, the generalizability of the results is limited by this being a single site and single country study, confined to TKA and THA patients. Future studies should thus be implemented to test if these results can be replicated.
